# Two Strains of Male-Killing *Wolbachia* in a Ladybird, *Coccinella undecimpunctata*, from a Hot Climate

**DOI:** 10.1371/journal.pone.0054218

**Published:** 2013-01-21

**Authors:** Sherif Elnagdy, Susan Messing, Michael E. N. Majerus

**Affiliations:** 1 Department of Genetics, University of Cambridge, Cambridge, United Kingdom; 2 Botany Department, Faculty of Sciences, Cairo University, Giza, Egypt; 3 Division of Psychiatric Statistics, Department of Biostatistics and Computational Biology, University of Rochester, Rochester, New York, United States of America; University of Poitiers, France

## Abstract

Ladybirds are a hot-spot for the invasion of male-killing bacteria. These maternally inherited endosymbionts cause the death of male host embryos, to the benefit of female sibling hosts and the bacteria that they contain. Previous studies have shown that high temperatures can eradicate male-killers from ladybirds, leaving the host free from infection. Here we report the discovery of two maternally inherited sex ratio distorters in populations of a coccinellid, *Coccinella undecimpunctata*, from a hot lowland region of the Middle East. DNA sequence analysis indicates that the male killing is the result of infection by *Wolbachia*, that the trait is tetracycline sensitive, and that two distinct strains of *Wolbachia* co-occur within one beetle population. We discuss the implications of these findings for theories of male-killing and suggest avenues for future field-work on this system.

## Introduction

Male-killing bacteria are ultra-selfish maternally transmitted endosymbionts whose spread within host populations depends on the damage they do to these hosts [1]. Although some male-killers act late in development, here we focus on male-killers that act early in development and whose dynamics do not entail significant levels of horizontal transmission [2]. All known agents of early male-killing are bacterial, with these agents being taxonomically diverse (including *Rickettsia* [3], Flavobacteria [4], *Spiroplasma*, [5], [6], *Wolbachia* [7] and γ-Proteobacteria [8]. The diversity of the agents associated with male-killing sets the male-killing strategy apart from the other ultra-selfish manipulations of host reproduction, most of which are caused by *Wolbachia* [9].

Early male-killers have been found in a wide range of insect species, including Coleoptera, Lepidoptera, Diptera, Hemiptera and Hymenoptera [10]. Some groups, such as aphidophagous coccinellids, milkweed bugs and nymphalid butterflies, particularly of the genus *Acraea,* are especially prone to invasion [11].

Three characteristics of aphidophagous ladybirds make them prone to invasion by male-killers [12]. First, they lay eggs in tight clutches, predisposing them to strong interactions among sibling larvae [13]. Second, ladybirds are highly cannibalistic [14], with neonate larvae habitually consuming any unhatched eggs in their clutch, whether these are viable or not [13]. The potential for sibling egg cannibalism has imposed selection for embryos to develop and hatch rapidly [13]. As a result, neonate larvae are poorly resourced and show high mortality from starvation when they fail to find and subdue their first aphid prey [9]. In egg clutches laid by females infected with male-killing bacteria, male eggs fail to hatch and so are available to be eaten by infected female siblings, which thereby gain significant extra resources before they disperse to find aphid prey. They are, therefore, able to search for longer and subdue larger prey than are larvae from uninfected clutches [15], [16], [17]. Finally, the aphid prey of ladybirds is highly ephemeral due to rapid population increases and crashes [18]. Thus, ladybird larvae are often confronted with local resource scarcity, thus magnifying the benefit of sibling cannibalism.

Of the ladybirds possessing these traits – laying eggs in clutches, exhibiting sibling cannibalism, and feeding on aphids – about half of those surveyed (13 of 30) have been found to be infected with male-killers. Conversely, none of 12 surveyed species lacking one or more of these traits has been found to be infected with male-killing endosymbionts [19]. However, it should be noted that Weinert *et al.*, 2007 [20], assaying 21 species of European coccinellids, did find inherited symbionts of three clades (*Spiroplasma*, *Rickettsia* and *Wolbachia*) from *Chilocorus bipustulatus* and *Halyzia sedecimguttata*, both of which lack one or more of the three characteristics. However, in neither species was the phenotype caused by the bacteria ascertained [19].

In addition to the behavioural and ecological prerequisites for invasion and persistence of male-killers, the environmental conditions must also be conducive. Specifically, high temperature may, by suppressing growth or killing the bacteria, prevent their spread within a host species [19]. For instance, *Drosophila bifasciata* can be cured of male-killing *Wolbachia* by culturing the flies at 26°C instead of 21°C [21]. Similarly, *Drosophila equinoxialis* could be cured of male-killing *Spiroplasma* by exposing eggs to relatively high temperatures (34–40°C) [22]. Using, artificially transferred *Spiroplasma* into *Drosophila melanogaster*, Sakaguchi and Poulson [23] showed that high temperature exposure produced a full cure from infection.

High temperature impacts on male-killers have been demonstrated in three species of coccinellids: *Adalia bipunctata* infected with *Rickettsia* [24], *Adalia decempunctata* infected with *Rickettsia* [25], and *Coleomegilla maculata* infected with *Flavobacterium* [26]. In these cases, egg hatch rates increased, either completely or partially, and sex ratios became less female biased, tending towards 1∶1, following high temperature treatment (25–30°C). Furthermore, molecular investigations confirmed the absence of the bacteria in temperature cured lines. In some cases, curing was partial, as in *A. decempunctata*, where *Rickettsia* was found in some male progeny, leading to the deduction that male death depends on the bacterial density in the host [25]. As a result of these experiments showing that high temperature can cure hosts of male-killer infections, it has been hypothesized that male-killers may be rare in hot climates [27].

In all aphidophagous coccinellid species previously tested, male-killing bacteria have been shown to be sensitive to high temperature [24], [25], [26]. Furthermore, all coccinellid populations found to date to be infected with male-killing bacteria have been from temperate or Mediterranean climates. This may result from research bias, or may be a consequence of the temperature sensitivity of male-killing bacteria, thus restricting the distribution of coccinellid-infecting male-killers to geographical regions lacking very high temperatures [27].

The mode by which coccinellids become infected with male-killing bacteria is not known. However, there is phylogenetic evidence to suggest that horizontal transmission of male-killing bacteria does occur, although probably very rarely [1]. This means that it is possible for one species of coccinellid to be invaded by two or more different types of male-killing bacteria. However, as the intracellular environment in which bacteria in a particular host species live and are vertically transmitted seems to be essentially the same, and as the bacteria employ the same strategy of ultra-selfish manipulation, the competitive exclusion principle would suggest that only a single male-killer should survive in a particular host population. Indeed, models of the invasion dynamics of early male-killers show that two male-killers cannot occupy the same population at equilibrium, unless there is some degree of male-killer suppression [28]. Randerson *et al*. [28] demonstrated that the ‘strongest’ male-killer: i.e. the most efficient in terms of high vertical transmission, low direct cost on infected females and high male-killing efficiency, would out-compete and exclude ‘weaker’ male-killers, unless the host can evolve resistance against the strong male-killer, which then allows the two to coexist in the same host population. Despite these theoretical findings, two or more male-killers have been recorded from some host populations without any indication that suppressor genes have evolved in the host. In the butterfly *Acraea encedon,* two strains of male-killing *Wolbachia* have been reported from Tanzanian populations [29]. In the coccinellid *Adalia bipunctata*, Majerus *et al*. [30] reported that four male-killers (a *Rickettsia*, a *Spiroplasma* and two distinct strains of *Wolbachia*) were found in a single sample collected from a single location in Moscow.

Here, we report the discovery of two male-killing strains of *Wolbachia* in a population of a coccinellid, *Coccinella undecimpunctata*, from hot regions of lowland Egypt and Jordan. This finding indicates that climate is less of a limiting factor for the distribution of male-killers in ladybirds.

## Materials and Methods

### Sample Collection and Culturing

200 individuals of adult *Coccinella undecimpunctata* L. were collected from Abo-Rawash, Giza, Egypt, in September 2004 and July 2005. An additional 50 individuals were collected in Amman, Jordan, by Professor T. F. Allawi, in October 2004. They were grouped in 10 individuals and housed in 9 cm stock Petri-dishes, and allowed to mate freely. Individual pairs were removed to clean dishes in which to lay eggs for the establishment of individual matrilines. Reproductive adults and larvae were allowed to feed on pea aphids (*Acrythosiphon pisum*), following methods described by Majerus and Kearns [31]. Eggs were harvested daily by removing adults to clean dishes, leaving the eggs *in situ*. Immature stages were reared in a controlled temperature room at 21°C and 16L: 8D. An artificial diet [32] was used to maintain non-reproductive adults. No specific permits were required for the described field studies. The samples location is not privately-owned or protected in any way. The field studies did not involve endangered or protected species.

### Phenotypic Indicators of Male-killing

Two phenotypic indicators of male-killing were assessed for each matriline: the egg hatch rate and the sex ratio of progeny. Once neonate larvae had dispersed, the eggs within each clutch were categorized as hatched, unhatched and yellow (which is indicative of there having been no embryonic development), or unhatched and grey (indicative of some embryonic development) [24]. Progeny sex ratios (given as proportion male) were obtained for each family by determining the sex of all progeny, using sex-specific ventral abdominal sternite traits, visualized microscopically under CO_2_ anaesthesia. Families were split into four categories: 1. Sex ratio (SR) = low egg hatch rate (<0.5) and all progeny female. 2. Incomplete sex ratio (iSR) = low egg hatch rate (<0.5) and a statistically significant female bias amongst progeny. 3. Normal (N) = high egg hatch rate (>0.5) and progeny sex ratio not significantly different from 1∶1. 4. Not ascertained (?N) = high egg hatch rate (>0.5) and progeny sex ratio significantly different from a 1∶1 sex ratio.

In some instances, matrilines initially designed as SR or iSR subsequently showed increased hatch rates and sex ratio (proportion males), and these were described as revertant families [4]. In general, these lines were removed from the study stocks, unless otherwise stated. No obvious case of progressive sex ratio [33] was observed in the study stocks.

### Inheritance of the SR Trait

Female offspring of SR and iSR lines were crossed with males from N families, to test the inheritance of the sex ratio trait. Pairs and their offspring were treated as before.

### Susceptibility of the SR Trait to Antibiotics

The effect of tetracycline, a broad-spectrum antibiotic, on females showing the SR trait was investigated. If the causative agent of the sex distortion was a bacterium, tetracycline treatment should produce a partial [34] or full [24], [35] cure of the trait. Eggs were collected from three females from an SR line for up to three weeks. The egg hatch rates of these females were recorded. Once it had been confirmed from egg hatch rates that these females remained less than 50%, females were fed for two hours per day on a diet of golden syrup containing 10% w/v tetracycline, being otherwise fed on aphids [24]. The time of exposure to tetracycline was varied from 1–4 weeks for different females. Five females from normal (N), presumably uninfected lines were treated in the same way as controls. The egg hatch rates after treatment and the progeny sex ratios from the eggs laid before and after treatments were recorded. Subsequently, molecular assays on progeny from treated females were used to assess the presence or absence of bacteria. For two of the treated SR families, one female resulting from an egg laid after tetracycline treatment was mated to a male from an N line, and her progeny reared, with the sex ratio of progeny being recorded.

### Identification of the Male-killing Agent

Genomic DNA was extracted from adult ladybirds, following a fast protocol modified by Majerus *et al.* [36] from Walsh *et al.* [37]. Successes of extractions were tested using general insect primers (*COI* gene) C1-J-1751f and C1-N-2191r [38], to check for the presence of ladybird DNA.

Modified versions of the standard PCR protocols for male-killers [1], [3], [36], [39] were used to assay two females from matriline B (SR) for bacteria. *16S rDNA* general eubacterial primers 27f and 1495r [40], were used. Product bands were sliced from the agarose gel and purified using Qiaquick PCR purification kit (Qiagen), and directly sequenced using dye-labelled terminators in a cycle-sequencing reaction (Applied Biosystems). The products were then visualised on an ABI 377 automated sequencing machine. Initial identification of the sequence was established through a BLAST search [41].

### Relation between the SR Trait and the Putative Causative Agent

Following identification of *Wolbachia* as a candidate male-killer, samples from all matrilines, irrespective of their sex ratio status, were assayed for *Wolbachia* using primers *wsp*81F and *wsp*691R [42] (Table 1). From the first Egyptian sample (2004) 39 SR or iSR females from three SR lines, 16 N or ?N females and five N males from three N lines were assayed. In addition, ten females produced by an SR line female before administration of tetracycline and eight females and two males produced by females from this SR line after tetracycline treatment were assayed. From the second Egyptian sample (2005) and the Jordanian sample, 28 SR or iSR line females, ten N females and five N line males were assayed. In addition, five females produced by SR line females before administration of tetracycline and six females and two males produced by SR line females after tetracycline treatment were assayed. DNA samples extracted from two previously known *Wolbachia*-infected *Adalia bipunctata* [30] were used as positive controls, while negative controls were prepared using the same PCR premix, except that sterile distilled water replaced the genomic DNA. PCR reactions were carried out in a Techne Progene PCR machine with a heated lid. All PCR cycling conditions were as described by Majerus [43]. PCR products were then run on a horizontal 1% agarose gel, beside an appropriate marker containing DNA fragments of known size (1 kb DNA ladder). Following electrophoresis, the gels were visualised and photographed using a Biogene UV trans-illuminator.

**Table 1:** The number of individuals molecularly assayed for *Wolbachia* using primers *wsp*81F and *wsp*691R.

Sample	Linename	Linestatus	♀	♂	Total
Egyptian2004	A	SR	13	0	39 SR♀, 16 N♀, 5 ♂
	B	SR	13	0	
	F	iSR	13	0	
	D	N	6	2	
	E	N	5	2	
	G	N	5	1	
Egyptian2005	E1	SR	5	0	15 SR♀, 3N♀, 2 ♂
	E2	SR	5	0	
	E3	SR	5	0	
	E4	N	4	2	
Jordanian2005	J2	SR	5	0	13 SR♀, 6N♀, 3 ♂
	J3	iSR	4	0	
	J4	SR	4	0	
	J1	N	3	2	
	J5	N	3	1	

### Phylogenetic Analysis

**Figure 1:**
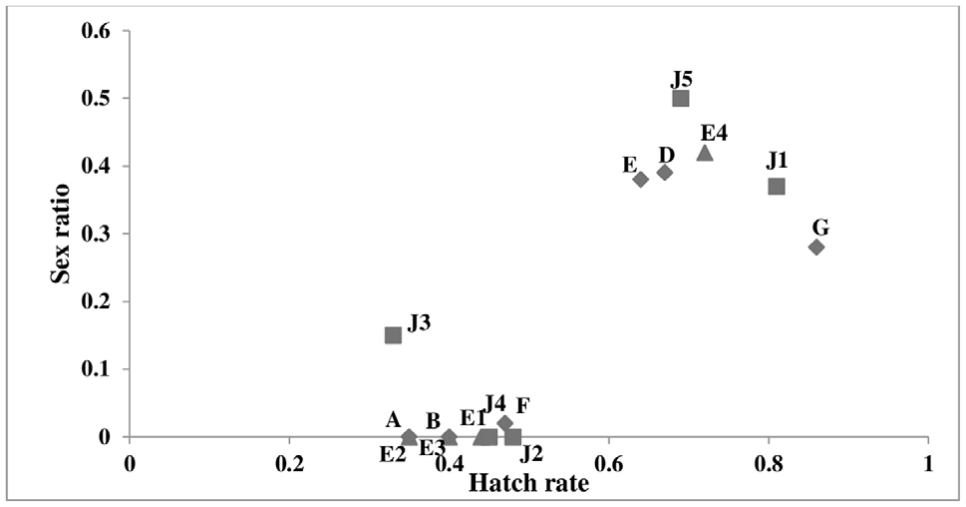
Egg hatch rates and progeny sex ratios (± standard errors) of matrilines of *C. undecimpunctata*. Hatch rate was measured by comparing the number of hatched eggs (H) to the total number of laid eggs (hatched+grey+yellow). The matrilines are labelled as follows: Egypt 2004 - A-G with diamond symbol; Egypt 2005 - E1–E4 with triangle symbol; and Jordan - J1–J5 with square symbol.

DNA from SR lines was amplified with *Wolbachia* specific primers *wsp*81F and *wsp*691R [42] for the *wsp* gene. Products were sequenced as described above for the *16S rDNA* gene and the resultant partial sequence was subjected to a BLAST search [41]. The sequences were then manually aligned with a selected group of *wsp* sequences (see Fig. 1 for details), including those known to cause male-killing (including the only known two male-killing *Wolbachia strains* in ladybirds, which are in *Adalia bipunctata*), at least one strain causing each of the other reproductive manipulations of hosts, and those showing closest homology to the sequence under investigation. Phylogenetic analysis was performed using Mega version 3.1 [44]. Phylogenetic trees were constructed using maximum parsimony and support was obtained from 500 bootstrap replicates; seed 64238 (program Mega 3.1). Gaps or missing data were dealt with by complete deletion.

### Statistical Analyses

Using StatXact 8 software, Fisher’s exact test was used to test the significance of deviation from 1∶1 sex ratios among family progeny. Bonferroni correction was used to correct for multiple comparisons among the different families. The association between the F1 and F2 sex ratios and the F1 sex ratio and hatch rate were evaluated using a simple linear regression model to report slope, associated error of estimate and P-value, as well as the correlation coefficient. The heterogeneity between lines was investigated using a Chi-square test to test the differences in proportions of sex ratio and hatch rates. Sex ratio changes subsequent to antibiotic treatments were also evaluated with a simple linear model which looked at information that we combined over different observations periods.

## Results

### Phenotypic Results

Sixteen matrilines were obtained from the Egyptian and the Jordanian collections, one of which failed to produce sufficient offspring for analysis. From these lines, nine lines were designated as SR lines, having low egg hatch rates and absence of male progeny (3 from Egyptian 2004 collection, 3 from the Egyptian 2005 collection, and 3 from the Jordanian collection) (Table 2). The rest of were designated as normal lines due to their normal sex ratio (Figure 1).

**Table 2:** Progenic sex ratios and sex ratio status of F1 females of *C. undecimpunctata* of 2004 Egyptian sample (A-G), 2005 Egyptian sample (E1–E4) and Jordanian sample (J1–J5) lines.

Line	H	G	Y	Hatch rate	Progeny			Fisher’s exact test	Sex ratio status
					♀	♂	Sex ratio		
A	42	6	72	0.35	20	0	0	F = 14.4, d.f. = 1, p<0.001	SR
B	21	0	31	0.40	4	0	0	F = 2.22, d.f. = 1, p = 0.214	SR
D	82	2	38	0.67	25	16	0.39	F = 0.98, d.f. = 1, p = 0.374	N
E	90	4	47	0.64	34	21	0.38	F = 1.533, d.f. = 1, p = 0.250	N
F	67	4	71	0.47	42	1	0.02	F = 27.78, d.f. = 1, p<0.001	iSR
G	113	2	16	0.86	55	22	0.28	F = 7.341, d.f. = 1, p<0.01	N
E1	42	0	53	0.44	15	0	0	F = 10.12, d.f. = 1, p<0.01	SR
E2	63	2	115	0.35	22	0	0	F = 16.03, d.f. = 1, p<0.001	SR
E3	66	4	95	0.4	30	0	0	F = 22.62, d.f. = 1, p<0.001	SR
E4	94	7	30	0.72	36	27	0.42	F = 0.786, d.f. = 1, p = 0.478	N
J1	74	2	15	0.81	27	16	0.37	F = 1.403, d.f. = 1, p = 0.278	N
J2	21	0	23	0.48	3	0	0	F = 1.645, d.f. = 1, p = 0.4	SR
J3	52	2	104	0.33	16	3	0.15	F = 4.8, d.f. = 1, p = 0.025	iSR
J4	29	1	34	0.45	2	0	0	F = 1.222, d.f. = 1, p = 0.5	SR
J5	33	0	15	0.69	2	2	0.5	F = 0.185, d.f. = 1, p = 0.514	N

Hatch rate was measured by comparing the number of hatched eggs (H) to the total number of laid eggs (hatched (H)+grey (G)+yellow (Y)). Sex ratio is given as the proportion of male offspring. Significance p-value using Bonferroni correction was 0.01.

**Figure 2:**
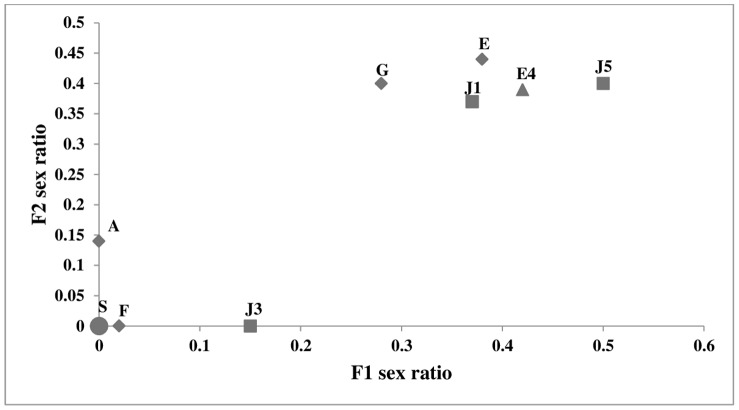
*C. undecimpunctata* matrilines females & F1 females sex ratio (proportion of male offspring) with standard error. Matrilines are labeled as in Figure 1. The letter “S” and the circle symbol represent all lines that have zero sex ratio in both F1 and F2 from the three collections (B, E1, E2, E3, J2 & J4).

Lines that had produced all or almost all female progeny in the F1 also did so in the F2, and lines with normal F1 sex ratios remained normal in the F2(Figure 2). The strong correlation between the F1 and F2 sex ratios (*r^2^* = 0.86, slope  = 0.93±0.11; *P*<0.0001) demonstrates essentially perfect maternal inheritance of the male-killing effect (Figure 2).

The F1 progeny sex ratios of all matrilines showed significant heterogeneity (χ^2^_11_ = 65, *P*<0.0001 )Furthermore, the progeny sex ratio was significantly correlated with egg hatch rate (*r* ^2^ = 0.68, slope  = 0.91±0.11, *P*<0.001), such that clutches with hatch rates less than 50% were made up almost entirely of females (Fig. 1).

### Effect of Tetracycline Treatment

The sex ratios of progeny produced before and after administration of tetracycline to females from an SR line are given in Table 3a. Families reared from F1 females produced by SR mothers after tetracycline treatment all produced high egg hatch rates and normal progeny sex ratios. The treatment resulted in an initial decrease in egg hatch rates, with hatch rates close to zero for two to five days (characteristic of ladybirds when treated with tetracycline) [45], followed by an increase in egg hatch rates to greater than 0.5. The sex ratio before antibiotic treatment was significantly different from that after antibiotic treatment in females 2 (p<0.05), 3 (p<0.01) and 4 (p<0.05), but not in females 1 (p  = 0.27) or 5 (p  = 0.91).

**Table 3:** Effect of tetracycline treatment on Egyptian lines (sex ratio is proportion of males to all offspring).

	Female	Period of treatment	Pre-treatmentegg hatch rate	Pre-treatmentprogeny	Sex ratio	Post-treatment egg hatch rate	Post-treatment progeny	Sex ratio	Sexes in F3	Sex ratio
**a**	1	1 week	0.36	8♀, 0♂	0	0.59	2♀, 1♂	0.33		
	2	3 weeks	0.34	18♀, 0♂	0	0.64	29♀, 20♂	0.41	24♀, 26♂	0.5
	3	4 weeks	0.32	10♀, 0♂	0	0.72	9♀, 10♂	0.52	6♀, 6♂	0.5
**b**	4	3 weeks	0.40	9♀, 0♂	0	0.62	21♀, 14♂	0.4	9♀, 8♂	0.47
	5	4 weeks	0.35	8♀, 0♂	0	0.70	2♀, 2♂	0.5	2♀, 2♂	0.5
**c**	N1	4 weeks	0.61	8♀, 5♂	0.8	0.60	2♀, 2♂	0.5		
	N2	4 weeks	0.77	5♀, 3♂	0.37	0.67	4♀, 3♂	0.43		
	N3	4 weeks	0.67	8♀, 4♂	0.33	0.70	5♀, 4♂	0.44		
	N4	4 weeks	0.80	7♀, 4♂	0.36	0.74	5♀, 3♂	0.37		
	N5	4 weeks	0.72	4♀, 3♂	0.42	0.69	6♀, 4♂	0.4		

a) Results from the 2004 Egyptian F1 females from SR lines. b) Results from two 2005 F1 females from line 3 (bearing a second male-killing strain). c) Results from 5 F1 females from the 2004 Egyptian N lines. Sexes in F3 refer to the progeny of one F2, post-treatment female mated to an unrelated male.

The sex ratio of progeny from SR females produced after tetracycline treatment increased rapidly, and converged upon 1∶1. Females from N lines fed on tetracycline showed no significant alteration of egg hatch rate or progeny sex ratio (Table 3b).

### Relation between *Wolbachia* Presence and the SR Trait

All SR females that had not been treated with tetracycline were found to be positive for the presence of *Wolbachia*. None of the females from N lines, or progeny of females from SR lines after tetracycline treatment, males from either these lines or N lines, or either of the negative controls were found to have *Wolbachia* present.

Moreover, while females produced by SR females before being treated with antibiotic tested positive for *Wolbachia,* none of these females and two males produced by these same SR females after tetracycline treatment did. Both negative control samples also tested negative.

### Phylogenetic Analysis

**Figure 3:**
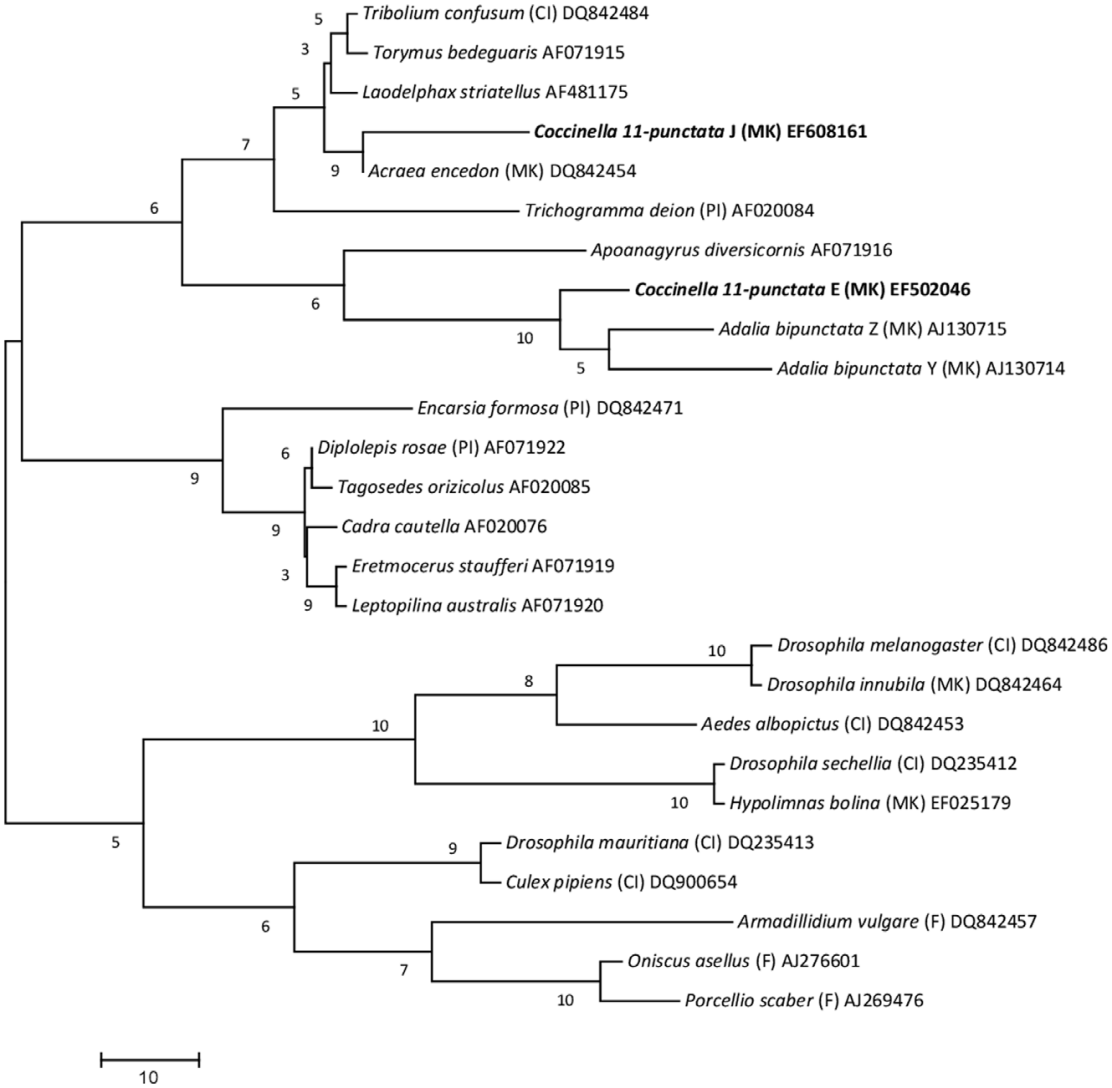
Phylogenetic tree of *wsp* DNA sequence data of *Wolbachia* hosted by different species. Maximum parsimony-based bootstrap analysis of different hosts of *Wolbachia* including the two *Wolbachia* strains of the *C. undecimpunctata*. Bootstrap values are indicated above the branches. *Wolbachia* strains are represented by the names of their host species, their phenotype where known (CI = cytoplasmic incompatibility; PI = parthenogenesis inducing; F = feminizing; MK = male-killing) and Genebank accession numbers. Suffix letters for *A. bipunctata* refer to the two different *Wolbachia* sequences, lodged by Hurst *et al.* (1999b). The *wsp* sequences found in this study are the *Coccinella 11-punctata* E (MK) EF502046 and *Coccinella 11-punctata* J (MK) EF608161 (in bold). *Wolbachia wsp* sequences used in constructing this tree were obtained from the Entrez Nucleotide database.

The analysis of the *16S rDNA* gene from females from the Egyptian 2004 SR matrilines showed that the male-killing causative agent may be a B-type *Wolbachia* (Accession number DQ993358). Phylogenetic analysis of this *wsp* sequence (Accession number EF502046) (Fig. 3) shows closest affinity to the two male-killing *Wolbachia* sequences from *A. bipunctata* reported by Hurst *et al.* [7]. This conclusion should be viewed with some caution because the *wsp* can undergo recombination between different *Wolbachia* strains, thus affecting phylogenetic patterns.

Analysis of the *wsp* gene revealed that our samples included two distinct strains of *Wolbachia* strains in our samples (Table 1). One strain (*wsp* Accession number EF502046) was found in three lines from Egypt in 2004 (A, B and F) and one line from Egypt in 2005 (line 1). A second *Wolbachia* strain (*wsp* Accession number EF608161) was found in two lines from Egypt in 2005 (lines 2 & 3) and three lines from Jordan (Lines 2, 3 &4). The sequence of this second strain differed from that of the first strain by 20.97% (of 515 bases) (Figure 3). The *wsp* sequence from the second strain is most similar to that of the U strain of *Acraea encedon*, with divergence of 15.13% (of 522 bp), including a central identical region of 387 bp.

On the basis of the limited data in this study, the prevalence of the first *Wolbachia* strain, which was only recorded from the Egyptian samples (sample size  = 50), is 0.4 (3 of 6 in 2004 and 1 of 4 in 2005). The mean fidelity (±se) of vertical transmission (calculated as: 1 - (number of males/number of females) [15] is estimated as 0.987±0.012. The second *Wolbachia* strain, which was recorded from both Egypt and Jordan, had an overall prevalence of 0.33 (combining 2 of 10 in Egypt and 3 of 5 in Jordan). For this second strain, the vertical transmission is estimated as >0.99 from Egypt and 0.85 from Jordan, giving a mean vertical transmission for this strain of 0.94±0.109. Overall, 60% (9 of 15) of the lines were infected with one of these male-killing *Wolbachia* strains.

## Discussion

The results show that *C. undecimpunctata* is a host to at least 2 different strains of male-killing B-type *Wolbachia*. The association between the *Wolbachia* infection and the SR trait was perfect. The *Wolbachia* was detected in females from all strains with significantly female-biased sex ratios and low hatch rates (SR and iSR), but from none of the strains with normal sex rations (N and ?N). One line (G) from the 2004 Egyptian sample had a significantly female biased sex ration among the F1, but had a high egg hatch rate (0.86). However, the F2 exhibited both a high hatch rate and normal sex ratio, and this line was found to be negative for *Wolbachia* infection. Thus, the F1 sex ratio bias may have been due to chance or to a factor other than a male killing endosymbiont (e.g., meiotic drive).

Both male-killing strains of *Wolbachia* identified here are antibiotic sensitive, as antibiotic treatment increased hatch rates of eggs laid by treated females, and restored a 1∶1 progeny sex ratio, and resulted in the production of offspring uninfected with *Wolbachia*. Moreover, the offspring of tetracycline-treated females produced normal progeny sex ratios.

Although the two *Wolbachia* strains found in *C. undecimpunctata* had identical 16S sequences, their *wsp* sequences were 20% divergent. Using the rapidly evolving *wsp* gene to infer relationships to other closely related strains, we find that one of the two strains in *C. undecimpunctata* is closely related to the two different but closely related male-killing *Wolbachia* strains that occur in the ladybird *A. bipunctata* [7]. Such a pattern could result from each beetle species having been invaded by closely related strains of *Wolbachia* that subsequently evolved male-killing within their new ladybird hosts. Alternatively, which is more likely, a male-killing strain may have jumped from one ladybird species to another via lateral transfer.

The other *Wolbachia* strain found in *C. undecimpunctata* is most similar to the male-killing *Wolbachia* in *A. encedon* [29]. This result supports previous hypotheses that horizontal transmission of male-killers may occur occasionally between hosts from different genera or even orders [7], [46], [47], [48]. Dyson *et al*. [48] suggested that some *Wolbachia* strains might specialise on particular host sex determination systems. The apparently close relationship between the second *C. undecimpunctata* strain and the U strain of *A. encedon* is thus striking, since most ladybirds, including *Coccinella*, have an XY sex determination system [13], while butterflies have a ZW sex determination system. If the same *Wolbachia* strain can cause male-killing in XY male beetles and ZZ male butterflies, this would suggest that some male-killing *Wolbachia* are generalists when it comes to sex determination systems. This possibility could be tested by transferring *Wolbachia* between *C. undecimpunctata* and *A. encedon*.

This is the first report of a male-killing endosymbiont in any coccinellid species from a hot climate. The daily high temperatures in Amman, Jordan and Giza, Egypt average ∼32°C and ∼34°C, respectively, during the hottest summer months, well above the temperatures shown to kill or debilitate male-killing endosymbionts from more temperate regions [24], [26], [49]. Thus, high temperature is not an insurmountable barrier to infection of coccinellids by male-killing bacteria. Further work on aphidophagous coccinellids from hot climates is likely to extend the number of cases of male-killing infection in this family of beetles. In some coccinellids, such as *Harmonia axyridis* infected with *Spiroplasma*, the temperature required to kill male-killing infections is close to the critical temperature at which coccinellids can survive, making cure of the SR trait by temperature difficult [43]. It would be interesting to determine whether the *Wolbachia* that infects Egyptian *C. undecimpunctata* can be cured by heat treatment without adversely affecting the beetles. Whether or not this is the case, the *Wolbachia* strains found in *C. undecimpunctata* from Egypt and Jordan provide an opportunity to explore the evolution of high-temperature tolerance in *Wolbachia*. The present findings also indicate that the ecology and evolution of ladybird beetles from hot climates may be as likely to be affected by male-killing endosymbionts as those from more temperate regions.

In previous assays of *C. undecimpunctata* samples from Britain (n  = 47), male-killers have not been found [19]. This may be because of low prevalence and incomplete ascertainment, or it may be because male-killers are absent from British populations of this species. If the latter is the case, this might be due to lack of invasion opportunity, or the result of inherent resistance, making British *C. undecimpunctata* an unsuitable host for male-killers. Microinjection of one of the male-killing *Wolbachia* that occurs in *C. undecimpunctata* in the Middle East, into British individuals could be used to test this latter possibility.

This is the first confirmed case of a heritable male-killing bacterium having been reported from the genus *Coccinella*. This is noteworthy because the majority of species in this genus are aphidophagous and have the ecological characteristics that should make them liable to infection by such bacteria. Despite this, in previous assays of 19 collections from seven species of *Coccinella* from temperate or Mediterranean climates (including two previous samples of *C. undecimpunctata* from England) (M. Majerus, unpubl. data), no male-killers have been detected. The results herein suggest that either further testing of members of this genus would be worthwhile or might reveal that *Wolbachia* in this genus may not only tolerate, but actually require high temperature for expression and transmission.

The results to date do not allow resolution of whether *C. undecimpunctata* was invaded by a single strain of *Wolbachia* that has subsequently diverged, or was invaded by two already different strains of *Wolbachia*. This question might be resolved by analysis of mtDNA variability associated with hosts of each *Wolbachia* strain. This is the third finding of more than one male-killing bacterial strain occurring sympatrically in the same host. This instance is similar to that in *A. encedon* in Tanzania, where two different strains of *Wolbachia* coexist [29]. The similarity to *A. bipunctata*, is less clear, for here, in addition to two strains of *Wolbachia* coexisting, two other phylogenetically disparate male-killers occur [30].

Randerson *et al*. [28] have tried in their model to explain the observed co-existence of multiple male-killing strains in the same host populations. They proposed the evolution and spread of a costly resistance gene in the host that should weaken the resident (the stronger) male-killer, although this may mean that both the resistance gene and the male-killer may be lost form the population. Then the weaker male-killer may spread at the expenses of the stronger male-killer, since it is tolerant to the effect of the evolved host resistance gene. As a result, the frequency of the stronger male-killer will decrease and in turn the frequency for the resistance gene will decrease as well. Thereafter, the frequency of the stronger male-killer may increase again, in a cyclical manner in response to the resistance gene. Such frequency dependent selection should allow stable coexistence of multiple male-killers in the same host population. However, the cause of male-killers coexistence in a population seems unconfirmed, especially no resistance genes have been reported in *C. undecimpunctata*.

Three scenarios seem possible. First, the cases of multiple male-killer existences in a host population may be the result of independent allopatric invasions by different strains of male-killer, followed by migration such that the different male-killers become sympatric. While this is tenable for the two coccinellid cases, for coccinellids can be highly dispersive [13], it seems to be less tenable for *A. encedon*, which is a highly colonial species showing low female dispersal. Moreover, in *A. bipunctata*, Tinsley [50] has shown that prevalence of two of the male-killers in this species (*Rickettsia* and *Spiroplasma*) show correlations with environmental factors in Scandinavia, suggesting long-term persistence.

Second, the Randerson *et al*. [28] model may be essentially correct, but the approach to male-killer prevalence equilibrium frequencies may be extremely slow, such that in those host populations that harbour two or more male-killers, equilibrium frequencies have yet to be reached, and the competitive advantage of the ‘strongest’ male-killer has yet to be manifest in the decline and elimination of ‘weaker’ male-killers. While this may be the case, if it is, it makes the prediction of competitive exclusion in the case of male-killers of little value in the field.

Finally, it is possible that biotic and abiotic factors affect the level of the three principle parameters that control male-killer invasion and prevalence (vertical transmission efficiency, direct effect of infection on females, fitness compensation) in coccinellids (1) in such a way that there is no consistently ‘strongest’ male-killer. Here, if the relative fitnesses of different male-killers oscillate such that the ‘strongest’ is first one strain and then the other, coexistence may be maintained for very long periods of time, particularly if there was a mechanism by which male-killer fitness was inversely related to prevalence.
